# Failure of Fondaparinux in Autoimmune Heparin-Induced Thrombocytopenia

**DOI:** 10.1055/s-0040-1713175

**Published:** 2020-10-20

**Authors:** Michelangelo Sartori, Benilde Cosmi

**Affiliations:** 1 Cardiovascular Department, Angiology and Blood Coagulation Unit, S. Orsola-Malpighi University Hospital, Bologna, Italy

**Keywords:** heparin-induced thrombocytopenia, rivaroxaban, deep vein thrombosis, fondaparinux

## Abstract

Heparin-induced thrombocytopenia (HIT) is an immune adverse reaction to heparin that is associated with life-threatening thrombotic complications. More rarely, HIT may begin after stopping of heparin or after flushes of heparin (autoimmune HIT). Fondaparinux has been proposed as a candidate treatment for HIT, but there are few data on its use in autoimmune HIT. An 86-year-old man with a history of diabetes mellitus, arterial hypertension, and hypercholesterolemia was admitted to our hospital for carotid endarterectomy. During surgery, only one heparin dose of 5,000 U was used. Platelet count started to decrease on the 11th day after surgery. Since the patient was not receiving heparin treatment/prophylaxis, HIT was not suspected. On day 19, platelet count was 61 × 10 ^3^ /μL, and the patient was investigated for a diagnosis of HIT. Immunoglobulin (Ig)-G-specific enzyme-linked immunosorbent assay (ELISA) was positive and HIT was confirmed by a platelet aggregation test; fondaparinux 5 mg once a day was started. During fondaparinux treatment, platelet count did not increase and a lower leg deep vein thrombosis occurred. Fondaparinux was stopped and rivaroxaban 15 mg twice a day was started. Platelet count returned to base line after 10 days from fondaparinux withdrawal. There was no thrombotic event or bleeding complication during rivaroxaban treatment. Anecdotal evidence suggests risk of failure of fondaparinux treatment for autoimmune HIT and supports the use of rivaroxaban for treatment of HIT, justifying larger studies.

## Introduction

Heparin-induced thrombocytopenia (HIT) is a prothrombotic condition that is associated with increased in vivo thrombin generation that needs to be treated with nonheparin anticoagulants. 1 In the majority of cases (typical-onset HIT), after a mean of 4 to 6 days of heparin exposure, immunoglobulin (Ig)-G antibodies against platelet factor 4 (PF4) bound to heparin develop. This is followed by the onset of the platelet count fall, and finally by thrombosis. 2 More rarely, HIT may follow the exposure to heparin “flushes” or after stopping heparin. Such condition has been described and classified as “autoimmune HIT.” 3 Often, autoimmune HIT patients have severe thrombocytopenia that may last for several weeks, and this is not a contraindication to anticoagulant therapy. 3 Patients with autoimmune HIT require nonheparin anticoagulants, high therapeutic levels of anticoagulation are needed to control such hypercoagulable state. 3 Recent guidelines recommend the use of danaparoid, argatroban, fondaparinux, or a direct oral anticoagulant for HIT treatment. 4 Fondaparinux is commonly used in every day clinical practice for clinically suspected and confirmed acute HIT. In a German registry, no thrombotic complication occurred in HIT patients treated with fondaparinux, 5 whereas some case reports have shown failure of fondaparinux anticoagulation for HIT with evidence of in vivo cross-reactivity HIT antibodies. 6 7 Recently, rivaroxaban, an oral direct factor Xa inhibitor, has been proposed as a candidate for treatment of HIT because it seems to have a better safety profile than the aforementioned agents and it is administered orally by fixed dosing. 8 As suggested by several case series, the administration of rivaroxaban may be adequate for thrombosis treatment and normalization of platelet count in patients with HIT 9 and with autoimmune HIT. 3 6 Here, we report a case of autoimmune HIT, in which a new thrombosis occurred during fondaparinux treatment, whereas rivaroxaban was not associated with thrombotic events or bleeding complications.

## Case Description

**Fig. 1:**
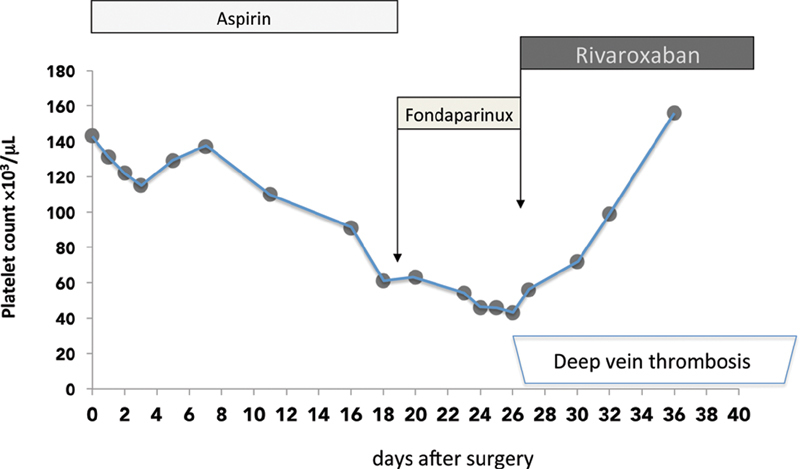
Trend in platelet counts after carotid endarterectomy (D0: day 0). On D0 a bolus of heparin dose of 5,000 U was used, 27 days afterwards (D27) a thrombosis confined to the internal gastrocnemius vein and to the soleal vein occurred (deep vein thrombosis). Fondaparinux: fondaparinux 5 mg once a day, Rivaroxaban: rivaroxaban 15 mg twice a day.

An 86-year-old man (weight: 74 kg) with a history of diabetes mellitus, arterial hypertension, hypercholesterolemia was admitted to our hospital for carotid endarterectomy. He was on therapy with ramipril 5 mg once a day, aspirin 100 mg once a day, amlodipine 10 mg once a day, omeprazole 20 mg once a day, and simvastatin 40 mg once a day. His medical history included a previous ischemic stroke and the presence of an infrarenal abdominal aortic aneurysm (diameter: 4.2 mm) and a 70% stenosis of left internal carotid. During carotid endarterectomy a bolus of unfractionated heparin (UFH), dose of 5,000 U, was used. Aspirin was continued during and after surgery. No prophylactic low molecular weight heparin was administered after surgery. As shown in the figure, in the morning before surgery his platelet count was 143 × 10 ^3^ /μL (day 0: D0), platelet count decreased to 115 × 10 ^3^ /μL on D3. On D5, platelet count increased, and reached 138 × 10 ^3^ /μL on D7. Platelet count was not repeated till D11 when a decrease was observed (110 × 10 ^3^ /μL). Since the patient was not receiving heparin treatment/prophylaxis, HIT was not initially suspected. On D16, platelet count was 91 × 10 ^3^ /μL, the pretest clinical score (4 T’s) for the diagnosis of HIT 10 was 4, but HIT was still not suspected. On D18, the platelet count declined further to 61 × 10 ^3^ /μL and the patient was therefore investigated for a diagnosis of delayed onset and persisting (autoimmune) HIT. A whole leg ultrasound did not show any deep vein thrombosis (DVT), 4T's score was 5 and, on D19, HIT was diagnosed on the basis of the presence of HIT antibodies. An IgG-specific ELISA (PF4-enhanced IgG, Immucor GTI Diagnostics, Inc.) was strongly positive (OD = 2.359). The presence of HIT was confirmed by a platelet aggregation test. Platelet aggregation was performed using a four channel Chrono-Log platelet aggregometer (model 540, Chrono-Log Corp., Havertown, Pennsylvania, United States): 10 μL of UFH (1 and 100 IU/mL final concentration) was added to the cuvette and the aggregation response was monitored for 20 minutes. 11 The presence of HIT was confirmed if the aggregation was >20% with 1 IU/mL UFH and completely inhibited or <20% with 100 IU/mL UFH. The patient's serum induced rapid activation of platelets from two donors in the presence of 1 IU/mL UFH, as well as in the absence of UFH, that was inhibited with 100 IU/mL UFH. He had renal insufficiency: serum creatinine was 1.2 mg/dL (estimated creatinine clearance with Cockcroft–Gault formula was 46 mL/min), and fondaparinux treatment was started. Given the absence of thrombosis and the presence of chronic kidney disease stage III, the dose of 5 mg once a day was used, aspirin was stopped. Since there was no clinical sign of venous/arterial thrombosis, the patient was discharged from the Hospital on D23. A close follow-up by our outpatient clinic was performed. During fondaparinux treatment, platelet count slightly decreased: being 46 × 10 ^3^ /μL on D24 and D25, and 43 × 10 ^3^ /μL on D27. On D27, he complained of calf pain and swelling, a whole leg ultrasound revealed a thrombosis confined to the internal gastrocnemius vein and to the soleal vein in the symptomatic leg, that is, isolated distal DVT. Fondaparinux was stopped and rivaroxaban 15 mg twice a day was started. As shown in Fig. 1 , the platelet count returned to baseline by 10 days following fondaparinux withdrawal. After 20 days since fondaparinux withdrawal, the patient was asymptomatic and the whole leg ultrasound showed recanalization of the calf DVT, rivaroxaban dosage was reduced to 20 mg once a day, the gel immunoassay was still positive for PF4-heparin complexes. After 3 months, rivaroxaban was stopped, whole-leg ultrasound did not show any distal DVT or any proximal DVT and the gel immunoassay gave a negative result. In summary, the patient completed a total of 3 months of rivaroxaban therapy with no recurrent thrombotic or bleeding complications. During follow-up (1 year), no arterial, neither venous thrombosis occurred.

## Discussion

Our experience suggests that fondaparinux should be used with caution in autoimmune HIT and, consistent with previous reports, rivaroxaban may be safely used to prevent new thrombosis in this hypercoagulable condition.

A recent German multicentre observational study showed no thrombotic complication in patient treated with fondaparinux 5 supporting the use of fondaparinux in the everyday clinical practice for the treatment of suspected and confirmed HIT. 12 Several limitation of the aforementioned study should be acknowledged: the study was retrospective, HIT was not confirmed in several patients, and the choice of the anticoagulant agent was left to the decision of the physician in charge. On the contrary, a retrospective study in English patients 13 found a 16% thromboembolic rate in patients treated with fondaparinux for HIT. Here, we report a case of autoimmune HIT refractory to fondaparinux. Patients with autoimmune HIT seem to be at higher thrombotic risk than patients with HIT and often require a higher and stable therapeutic levels of anticoagulation. Since the presence of chronic kidney disease and since no thrombosis was found, fondaparinux at a dose of 5 mg was used. It should be noted that the dose suggested for acute thromboembolism therapy is higher than the dose we used (5 vs. 7.5 mg), and this may explain the failure of fondaparinux in the present case. Moreover, it has been shown that fondaparinux may, although infrequently, cross-react with antiheparin PF4 antibodies, and at least theoretically provoke or exacerbate HIT. We did not measure fondaparinux cross reactivity with HIT antibodies, thus we can only speculate the cause of fondaparinux failure in the present case. It has to be noticed that, despite platelet started to decrease 11 days after surgery, HIT was suspected only after 16 days and anticoagulant treatment was started after 19 days. This delay may have lead to higher thrombotic burden and favored the occurrence of a new thrombosis.

The efficacy of rivaroxaban, an oral, direct factor Xa inhibitor, in the treatment of venous thromboembolism has been clearly established. 14 Rivaroxaban does not show any interaction with PF4 or anti-PF4/heparin, 15 and there is growing evidence that rivaroxaban can be an alternative anticoagulant agent in HIT, so far more than 100 patients with HIT have been treated with such agent with low thromboembolic and bleeding complications. 9 During the past 4 years, we have already used rivaroxaban in other two cases of HIT, one has already been described, 16 another was an HIT associated with DVT after orthopaedic surgery. We used rivaroxaban for HIT because it has been shown to be efficacious as single agent in the acute phase of DVT without a short initial parenteral anticoagulant treatment and it allowed us to treat the patient at home. We did not observed thromboembolic complications during rivaroxaban treatment in these three patients. It has to be noted that all the three patients whom we treated with rivaroxaban had HIT associated with thrombosis limited to the vein of lower leg. In our opinion, these patients may be a lower risk than patients with HIT associated with pulmonary embolism or arterial thrombosis.

Autoimmune HIT is caused by platelet-activating anti-PF4/polyanion antibodies that are able to activate platelets in the absence of heparin. 3 Autoimmune HIT encompasses several clinical conditions, including delayed-onset HIT, spontaneous HIT syndrome (HIT without proximate heparin exposure), flush heparin HIT (HIT induced by exposure to heparin flushes), fondaparinux-associated HIT (HIT triggered by exposure to fondaparinux), and is associated with persistent thrombocytopenia and often with disseminated intravascular coagulation. 3 This condition requires treated with consistently therapeutic levels of anticoagulation. Our patient clearly had a diagnosis of autoimmune HIT based on the clinical picture of delayed onset and persisting thrombocytopenia following a single-intraoperative UFH exposure, as well as the laboratory demonstration of patient's serum-induced platelet aggregation that occurred even in the absence of heparin but which was inhibited by high concentrations of heparin.

Rivaroxaban is one of the treatment options recommended for treating the hypercoagulability state associated with autoimmune HIT. 3 Indeed, at least seven cases of autoimmune HIT 6 9 17 18 19 have been successfully treated with rivaroxaban. In contrast, several reports 6 7 17 have highlighted unsuccessful outcomes when fondaparinux was used as the first therapeutic agent for autoimmune HIT. There are at least two explanations why rivaroxaban, given 15 mg twice a day, may be superior to fondaparinux in treating autoimmune HIT. First, twice-daily dosing of rivaroxaban versus once-daily fondaparinux dosing may better achieve persistent levels of therapeutic anticoagulation, and control of severe HIT-associated hypercoagulability. Second, autoimmune HIT may be a high-risk situation for fondaparinux cross-reactivity, a problem not seen with rivaroxaban.

## Limitations and Conclusion

Some limitations of our report should be acknowledged. First, we were not able to determine whether fondaparinux cross-reactivity was a potential explanation for treatment failure in our patient with autoimmune HIT. Second, we cannot exclude the possibility that the explanation for fondaparinux failure was the dose administered (5 mg), which was lower than the usual dose (7.5 mg) recommended for treatment of acute HIT-associated thromboembolism. However, it should be noted that the Food and Drug Administration (FDA) recommends that fondaparinux be used with caution in patients with creatinine clearance of 30 to 50 mL/min, and our patient had additional considerations (advanced age and concomitant antiplatelet medications). 20 Thus, the 5mg dose we gave seemed appropriate for initial treatment in our 86-year-old male patient with chronic renal disease. Despite the additional limitation of a single case report, our observations are consistent with emerging clinical experience and supports the intriguing hypothesis that rivaroxaban may be a superior option over fondaparinux for treating autoimmune HIT.
